# Hospital Festivals, Meetings, &c.

**Published:** 1895-11-09

**Authors:** 


### HOSPITAL FESTIVALS, MEETINGS, &C.

### WESTMINSTER HOSPITAL.

The Westminster Hospital, which has recently been closed for necessary improvements and repairs, was opened for a general inspection on the part of governors and their friends on Friday last, of whom quite a number accepted the invitation of the committee and gathered in the board-room to meet the Duke of Westminster, the president of the hospital, who was present with the Duchess. The Dean of Westminster and Sir Rutherford Alcock were unable to attend.

Mr. Quennell, the secretary, read an address to the president, giving an account of the work which had been accomplished before the visitors started to see the wards for themselves. The greatest alterations being in the drainage, which has been thoroughly re-laid throughout, there is not much change to be noted in the general aspect of the wards. These have been, as well as the corridors, painted, an improvement in appearance as in sanitation, distemper having been the previous method of treatment of the walls. The colours chosen are harmonious and pleasant to the eye, and with the charming polished floors and the addition of handsome and very solid oak tables, with tiled tops of a warm shade in the centre of each ward, the general effect is most happy. It seems a pity that nothing can be done to alter the present unfortunate juxtaposition of ward lavatories and sculleries, but no doubt the best that could be done has been done with the limited space at disposal. More room is, of course, the great want at Westminster, and it must be difficult enough to make the most of what there is available to the best advantage of patients and nurses. The whole of the exterior of the hospital has also been painted and generally overhauled, and everything has been done to ensure the most perfect sanitary conditions possible. The nurses' sitting-room has been made a most pleasant and comfortable room, and we are glad to see there the beginnings of a library. People who are interested in nurses might do worse than give an occasional contribution towards this necessary adjunct to their comfort. The operation theatre has received many important additions in the way of fittings; especially the hot and cold water arrangements, sinks, &c., are admirable, and the steriliser after the pattern of that at St. Thomas's Hospital.

The appeal for funds issued when the present alterations were decided upon met with a liberal response, in all some £5,200 having been brought together from various sources, an amount that will cover the extraordinary expenditure incurred, and of which it is very satisfactory that about £200 is in the form of annual subscriptions.

Tea was provided for the visitors—amongst whom, by the by, might have been noticed many faces well known in the hospital world—in the board-room and nurses' day-room, and an organ recital was afterwards given by Dr. Bridge in the pretty little chapel, followed by a short commemoration service, the chapel having been first opened on All Saints' Day, 1886.

A cordial vote of thanks was passed to the Duke and Duchess of Westminster for their presence, a token in itself of the interest invariably shown by the president in the welfare of the hospital with which his name has been connected for twenty-five years.

